# Animals and SARS‐CoV‐2: Species susceptibility and viral transmission in experimental and natural conditions, and the potential implications for community transmission

**DOI:** 10.1111/tbed.13885

**Published:** 2020-11-04

**Authors:** Emma C. Hobbs, Tristan J. Reid

**Affiliations:** ^1^ Australian Centre for Disease Preparedness (ACDP) Commonwealth Scientific and Industrial Research Organisation (CSIRO) East Geelong VIC Australia

**Keywords:** companion animals, experimental animals, novel 2019 coronavirus, transmission studies, wildlife, zoonotic diseases

## Abstract

The current COVID‐19 global pandemic, caused by severe acute respiratory syndrome coronavirus 2 (SARS‐CoV‐2) of probable bat origin, has highlighted the ongoing need for a One Health response to emerging zoonotic disease events. Understanding the human–animal interface and its relevance to disease transmission remains a critical control point for many emerging zoonoses. Determination of the susceptibility of various animal species to infection with SARS‐CoV‐2 and the role of animals in the epidemiology of the disease will be critical to informing appropriate human and veterinary public health responses to this pandemic. A scoping literature review was conducted to collect, evaluate and present the available research evidence regarding SARS‐CoV‐2 infections in animals. Experimental studies have successfully demonstrated SARS‐CoV‐2 infection and transmission in cats, ferrets, hamsters, bats and non‐human primates under experimental settings. Dogs appear to have limited susceptibility to SARS‐CoV‐2, while other domestic species including pigs and poultry do not appear susceptible. Naturally occurring SARS‐CoV‐2 infections in animals appear uncommon, with 14 pets, 8 captive big cats and an unreported number of farmed mink testing positive at the time of writing (early July 2020). Infections typically appear asymptomatic in dogs, while clinical signs of respiratory and/or gastrointestinal disease tend to be mild to moderate in felines, and severe to fatal in mink. Most animals are presumed to have been infected by close contact with COVID‐19 patients. In domestic settings, viral transmission is self‐limiting; however in high‐density animal environments, there can be sustained between‐animal transmission. To date, two potential cases of animal‐to‐human transmission are being investigated, on infected mink farms. Given the millions of COVID‐19 cases worldwide and ongoing potential for further zoonotic and anthroponotic viral transmission, further research and surveillance activities are needed to definitively determine the role of animals in community transmission of SARS‐CoV‐2.

**Abbreviations**
ACE2: angiotensin‐converting enzyme 2
CDC: Center for disease control and preparedness
dpi: days post‐infection
MPRSS2: transmembrane serine protease
OIE: World Organisation for animal health
PPE: personal protective equipment
SARS‐CoV‐2: , severe acute respiratory syndrome coronavirus 2
WADDL: Washington Animal Disease Diagnostic Laboratory

## 1 INTRODUCTION

First detected in late 2019, the COVID‐19 pandemic caused by the novel severe acute respiratory syndrome coronavirus 2 (SARS‐CoV‐2) has since been declared a public health emergency of international concern, approaching eleven million human infections and causing over half a million human deaths by early July 2020 (Dong et al., 2020). The zoonotic virus was first identified in Wuhan, China, and the initial cluster of cases was initially believed to be linked to a live “wet” seafood and animal market, although results of back tracing activities have now called this into question (Letzter, 2020; Woodward, 2020). Phylogenetically related to SARS‐CoV‐1, which caused the disease SARS that emerged from China in 2003, SARS‐CoV‐2 is also believed to have emerged from a betacoronavirus circulating in rhinolophid (horseshoe) bats (Lau et al., 2020; Zhou et al., 2020b), which then made the species jump to humans, potentially by way of an intermediate animal host. Various animal species including snakes, turtles and pangolins have been proposed as the intermediate host (Andersen et al., 2020; Lam, Jia, et al., 2020; Lam, Shum, et al., 2020; Liu et al., 2020; Xiao et al., 2020; Zhai, et al., 2020; Zhang et al., 2020b), with no consensus yet being reached.

From the initial zoonotic spillover event in China, the virus quickly demonstrated efficient human‐to‐human transmission, with virus in respiratory droplets expelled by COVID‐19 patients during coughing, sneezing and talking reportedly remaining viable for multiple hours in aerosols and up to days on some surfaces (van Doremalen et al., 2020; Meselson, 2020). With billions of people around the world harbouring the virus, and the presumptive animal‐origin and high recombination rates of SARS‐coronaviruses (Stavrinides & Guttman, 2004), reverse spillover of SARS‐CoV‐2 from humans to animals (anthroponotic transmission) was surely inevitable.

Incalculable numbers of human–animal interactions occur each day in domestic, agricultural, research, recreational, educational, therapeutic, entertainment and wild settings. Over 470 million dogs and 370 million cats were kept as pets worldwide in 2018 (Bedford, 2020), with the USA, China and Russia the top three dog‐ and cat‐owning nations (Walden, n.d.). Over half of 27,000 surveyed households owned at least one pet in 2016, and increases in pet ownership are reportedly highest in China, India and Latin America (GfK, 2016). Worldwide, an estimated 70 billion terrestrial animals were raised and slaughtered for human consumption in 2018, with farm animal production the fastest growing agricultural sub‐sector and increases particularly expected to occur in developing countries (Ilea, 2009). Flourishing legal and illegal wildlife trades exist worldwide, with the global trafficking in illegal wildlife commodities including pangolin scales, elephant ivory, tiger bone and bear bile estimated at $7–23 billion USD in 2016 (TRAFFIC, 2020). Encroachment of humans into previously uninhabited areas for logging, cropping and urbanization can create diverse wildlife‐livestock‐human interfaces that represent critical points for cross‐species transmission and emergence of pathogens (Hassell et al., 2017). Over 60% of recently identified emerging disease events have been zoonotic—the majority of which have had a wildlife origin—and such events have been increasing significantly in recent times (Jones et al., 2008; Wang & Crameri, 2014).

As recent outbreaks of zoonotic diseases with pandemic and panzootic potential—including highly pathogenic avian influenza, SARS and Ebola virus—have demonstrated, an understanding of the human–animal interface and its relevance to disease transmission remains a critical control point. For the current pandemic, determination of the susceptibility of various animal species to infection with SARS‐CoV‐2 and the role of animals in the epidemiology of the disease are critical to informing appropriate human and veterinary public health responses.

Many simulation models have been developed to assess the likelihood of SARS‐CoV‐2 susceptibility in animal hosts. These studies aim to classify animal susceptibility or elucidate possible intermediate or reservoir host species based on the implied affinity of the species’ angiotensin‐converting enzyme 2 (ACE2) receptor‐binding domain sites for the SARS‐CoV‐2 spike protein. Results have indicated that species at highest risk of infection with SARS‐CoV‐2 include primates—particularly of the catarrhine (Old world) order (Damas et al., 2020)—and most of the carnivores, even‐ and odd‐toed ungulates, pangolins and scaly anteaters (Praharaj et al., 2020). Medium to high probability was ascribed to pangolins, rabbits and cats; lower probability to chickens and turkeys; and rats, mice and ducks were rated as low to very low (Praharaj et al., 2020; Zhao et al., 2020). Zhai et al. (2020, 2020) noted that pigs and dogs, and even cattle and sheep appear to have ACE2 proteins capable of acting as a receptor for viral entry, and the apparent low susceptibility of infection in these species is likely related to the relatively low levels of ACE2 expressed in the respiratory tract. Notably, the Damas et al. (2020) model predicted domestic cats, tigers and golden hamsters to be only medium risk, and ferrets low risk. Contrary evidence of natural and experimental infection in these animals (see below) suggests that the utility of these models as predictive tools may be limited.

Given the conflicting results of predictive modelling studies and the large numbers of journal articles, preprints, press releases and media reports of varying quality that have flooded the literature in recent months, definitive answers regarding the infectivity and clinical picture of SARS‐CoV‐2 in animals have been difficult to find. To this end, this study aimed to review the current literature to identify animal species that have been conclusively shown to be permissive to SARS‐CoV‐2 infection and transmission, and to collate the latest information regarding natural and experimental infections of the virus in animals, in order to assess the potential role of animals in community transmission of COVID‐19.

## 2 MATERIALS AND METHODS

This literature review was conducted using an exploratory scoping study approach, as its purpose was to rapidly map the broad state of knowledge regarding SARS‐CoV‐2 and animals from the heterogeneous types of available evidence, rather than to answer a clearly defined question (Arksey & O’Malley, 2005). We conducted successive daily scans of multiple electronic databases, preprint repositories, internet search engines, government and organizational websites, and media sources, and used different combinations of the following words and phrases to actively identify additional relevant publications: SARS‐CoV‐2, coronavirus, COVID‐19, nCoV‐19, animal, pet, cat, dog, ferret, hamster, tiger, lion, monkey, primate, bat, pangolin, snake, mink, “reservoir host”, “intermediate host”, transmission, susceptibility, infection. The searches covered all years and any publications in English and Dutch. We also searched reference lists from key reviews and articles to identify additional publications or sources of interest. Study titles and abstracts were screened, with full articles obtained and re‐evaluated for inclusion/exclusion under the following criteria:

1. *Inclusion criteria:* Epidemiological studies, case reports, commentaries, reviews, letters, editorials, preprints, government documents, media reports and blogs, published in English or Dutch between 31st December 2019 and 23rd June 2020, that were relevant under the following categories: (1) reports of natural SARS‐CoV‐2 infections in domestic or wild animal species; (2) experimental SARS‐CoV‐2 susceptibility, pathogenesis and/or transmission studies in animals; (3) discussions, predictions and/or modelling of animal reservoir and/or intermediate host species for SARS‐CoV‐2, and (4) general reviews or commentary about SARS‐CoV‐2 and animals.
2. *Exclusion criteria:* Any documents relating to SARS‐CoV‐2 vaccines or therapeutics, including nanobody and antibody therapy, and articles published in a language other than English or Dutch.

Based on the above criteria, a total of 351 documents were collated and evaluated for inclusion in this review: 78 journal articles; 79 preprints; 75 media articles (including newspaper and magazine articles); 113 official reports, documents and press releases; and 6 websites. Included references were sorted into the categories listed above. Data from experimental studies were entered into a Microsoft Word template by animal species. Case data on natural infections in animals were entered into a Microsoft Excel spreadsheet, from which summary tables were created.

## 3 RESULTS

### 3.1 Experimental susceptibility and transmission studies in animals

Numerous studies have evaluated the susceptibility of various animal species to infection with SARS‐CoV‐2 in experimental settings, for the purposes of animal model development and to inform understanding of the potential role of animals in the emergence and transmission of SARS‐CoV‐2 in the community. Experimentally infected bats have demonstrated characteristics of reservoir hosts, whereas ferrets appear to most closely mimic human infections and viral transmission dynamics, making them a highly suitable animal model for COVID‐19 studies.

Results of experimental species susceptibility and transmission studies are summarized in Table 1 and presented below.

**TABLE 1:** Characteristics of animal species experimentally infected with SARS‐CoV‐2

**Animal species**	**Clinical signs**	**Pathological changes on necropsy**	**Replication**	**Transmission to in‐contact animals**	**Antibody response**
Ferrets	+++	+++	+++	+++	+++
Cats	++	++	+++	+++	+++
Dogs	−	−	−	−	+
Hamsters	+	DU	+	++	++
Non−human primates	+	+++	++	++	++
Fruit bats	−	DU	++	++	++
Tree shrews	−	++	+	DU	DU
Chickens	−	−	−	−	−
Ducks	−	−	−	−	−

Note

Extent of each characteristic is indicated by – (not seen), + (to some extent), ++ (to a moderate extent) or +++ (to a large extent).

Abbreviation: DU, data unavailable.

#### 3.1.1 Ferrets

Ferrets are highly susceptible to SARS‐CoV‐2, with efficient virus replication occurring in the upper respiratory tract (nasal turbinates, soft palate and tonsils) from as early as two days post‐infection (dpi). Highest viral titres were observed in nasal washes, peaking at 4 dpi before dropping below detection limits at 10 dpi (Kim et al., 2020). Virus replication potentially also occurs in the digestive tract (viral RNA was detected in rectal swabs of infected ferrets) but does not appear to replicate in the lower respiratory tract, even in animals that were intratracheally inoculated. Antibodies were detectable in low quantities from 13 dpi, increasing substantially by 20 dpi (Shi et al., 2020). The virus is effectively transmitted via direct contact and to other in‐contact ferrets via the respiratory droplet route (Kim et al., 2020; Richard et al., 2020; Shi et al., 2020).

Clinical signs in infected ferrets included elevated body temperatures, loss of appetite, reduced activity and occasional coughing (Kim et al., 2020; Shi et al., 2020). The similarity of the clinical disease picture in infected ferrets and humans, and the efficient replication of SARS‐CoV‐2 in the ferret upper respiratory tract makes them a highly suitable animal model for evaluating antiviral drugs or vaccine candidates against COVID‐19.

#### 3.1.2 Cats

Cats are also susceptible to infection with SARS‐CoV‐2, with viral replication occurring in the upper respiratory tract following nasal inoculation (Shi et al., 2020). Peak oral and nasal viral shedding was recorded at 3 dpi in three directly inoculated cats, and on day 7 post‐exposure in two in‐contact cats (Bosco‐Lauth et al., 2020). Infectious virus is also excreted rectally (Shi et al., 2020).

Transmission occurs by both direct contact and indirectly via aerosols (Bosco‐Lauth et al., 2020; Shi et al., 2020). Experimental exposure has resulted in both subclinical and symptomatic infections, with juvenile cats (70 to 100 days old) reportedly more susceptible to severe clinical disease and death (Shi et al., 2020).

Antibody titres have been recorded in both experimentally inoculated and in‐contact cats (Bosco‐Lauth et al., 2020; Shi et al., 2020). Bosco‐Lauth et al. (2020) reported that infected cats (*n* = 3) developed significant neutralizing antibody titres that stayed stable or increased from 14 dpi up to 42 dpi. Following re‐challenge with SARS‐CoV‐2 at 28 dpi, a moderate increase in antibody titre was observed, and no virus was shed by any of the cats in the subsequent 14‐day period.

A study by Chen et al. (2020) suggests that cats have both a high frequency of cells expressing ACE2, and a high proportion of cells co‐expressing ACE2 and TMPRSS2 (transmembrane serine protease 2), both of which are targets for SARS‐CoV‐2 entry. These target cells are also widely distributed among organs within the feline digestive, respiratory and urinary systems, suggesting that cats may be susceptible to infection and transmission via several routes.

#### 3.1.3 Dogs

Dogs appear to have low susceptibility to SARS‐CoV‐2 following experimental inoculation. One study (Shi et al., 2020) intranasally infected five beagles with SARS‐CoV‐2, and collected samples over the subsequent two week period. Viral RNA was detected in three of the dogs’ rectal swabs between 2 and 6 dpi. No virus was detected in any oropharyngeal swabs taken from the dogs during the study period, nor in any organs or tissues on autopsy, and attempts at virus isolation were negative. Antibodies were detected in the sera of 2/4 experimentally infected dogs, but neither of two additional beagles that were housed in close proximity.

Chen et al. (2020) reported very low levels of co‐expression of ACE2 and TMPRSS2 target receptors in canine lung cells, as well as mutations in critical amino acid sequences in ACE2 receptors, which they suggested may be responsible for the low susceptibility of dogs to SARS‐CoV‐2 infection.

#### 3.1.4 Syrian golden hamsters

SARS‐CoV‐2 infections in Syrian golden hamsters appear to resemble features found in human patients with mild infections, with viral replication in the epithelial cells of the respiratory and gastrointestinal tracts following intranasal inoculation (Chan et al., 2020). Peak viral load 10^5^ – 10^7^ was detected in the lungs at around 2 to 3 dpi and high copy numbers of viral RNA continued to be detectable beyond 7 dpi, although no infectious virus was detected from 7 dpi.

Syrian hamsters developed mild clinical signs including weight loss, rapid breathing, and postural changes, with older hamsters (32–24 weeks old) exhibiting more pronounced and consistent weight loss than younger hamsters (6 months old) (Osterrieder et al., 2020). Other age‐dependent observations included an earlier and stronger influx of immune cells in lung tissue, and rapid lung recovery at day 14 post‐infection in younger hamsters. Older hamsters developed conspicuous alveolar and perivascular oedema indicative of vascular leakage (Osterrieder et al., 2020), similar to the more severe pathology observed in elderly COVID‐19 patients.

Transmission studies reported efficient viral transmission from infected to naïve in‐contact hamsters, resulting in similar pathology but not weight loss. All infected hamsters fully recovered and developed mean serum neutralizing antibody titre >1:427 by day 14 post‐infection (Sia et al., 2020), with higher antibody titres seen in younger animals (Osterrieder et al., 2020). Immunoprophylaxis with early convalescent serum achieved significant decrease in lung viral load but not in lung pathology (Chan et al., 2020).

#### 3.1.5 Non‐human primates

Experimental infection studies have been reported in a range of non‐human primate species, including rhesus macaques (*Macaca mulatta*), cynomolgus or crab‐eating macaques (*Macaca fascicularis*), common marmosets (*Callithrix jacchus)* and African green or vervet monkeys (*Chlorocebus aethiops)*.

An experimental study by Lu et al. (2020) determined that that rhesus macaques (*Macaca mulatta*) were the most susceptible to SARS‐CoV‐2 infection, followed by cynomolgus or crab‐eating macaques (*Macaca fascicularis*) and lastly common marmosets (*Callithrix jacchus)*. SARS‐CoV‐2 was detected in nasal, throat and anal swabs as well as blood from all three monkey species, with viral shedding from the upper respiratory tract peaking between 6 and 8 dpi (Denis et al., 2020).

Infections in all tested monkey species ranged from asymptomatic, to mild serous nasal discharge and transient or intermittent increases in body temperatures (Hartman et al., 2020; Rockx et al., 2020), to moderate respiratory disease with coughing, anorexia, postural changes and weight loss, and some radiographic chest abnormalities similar to those from human COVID‐19 patients observed (Lu et al., 2020; Munster et al., 2020). Hartman et al. (2020) also reported a transient decrease in lung tidal volume at 7 dpi. Post‐mortem examination of severely affected animals showed severe gross pathology in the heart, stomach and lower respiratory tract including diffuse interstitial pneumonia (Munster et al., 2020; Shan et al., 2020).

Viral replication was highest in lung tissue, but was also detected in ileum and tracheobronchial lymph nodes (Rockx et al., 2020). Viral shedding in oropharyngeal swabs peaked between 1 and 5 dpi, decreasing below detectable limits by day 9, with one study recording a second recrudescent period of viral shedding from respiratory and gastrointestinal tracts in all infected macaques including subclinical animals between 14–21 dpi (Hartman et al., 2020). Anal and rectal swabs tested positive in some animals up to 11 and 20 dpi, respectively (Munster et al., 2020; Shan et al., 2020). Higher levels of viral RNA were detected in nasal swabs of aged cynomolgus macaques compared to younger animals (Rockx et al., 2020). Viral RNA was detectable by RT‐PCR in peripheral blood between 2 and 10 dpi (Lu et al., 2020), and antibodies against the S1 domain and nucleocapsid proteins of SARS‐CoV‐2 were detectable from 14 dpi (Rockx et al., 2020).

Study authors concluded that the experimental non‐human primate species tested are permissive to SARS‐CoV‐2 infection, shed virus for pronged periods of time and display COVID‐19‐like disease, thereby providing suitable animal models for COVID‐19 studies (Munster et al., 2020; Totura et al., 2020).

#### 3.1.6 Fruit bats

Fruit bats (*Rousettus aegyptiacus*) intranasally inoculated with SARS‐CoV‐2 (*n* = 9) developed transient infection in the upper and lower respiratory tract, with virus replication detectable in the nasal epithelium, trachea, lung and lung‐associated lymphatic tissue. Infectious virus was isolated from the nasal epithelium and trachea of one bat at 4 dpi days after the infection of the experimentally inoculated bats. No clinical signs, elevated body temperatures, decreases in body weight or mortality were observed in any of the bats; these characteristics are consistent with those of a reservoir host (Schlottau et al., 2020).

#### 3.1.7 Tree shrews

Tree shrews (*Tupaia belangeris*) were considered as potential animal models for SARS‐CoV‐2 infection, as they are genetically similar to primates and have been used in biomedical research for animal models of viral infections including hepatitis B, influenza and Zika viruses (Zhou et al., 2020b). However, the one available study by Zhou et al. (2020b) reported limited viral replication in tree shrews (*n* = 24) intranasally inoculated with SARS‐CoV‐2. SARS‐CoV‐2 RNA was detectable in nasal, throat and anal swabs from 6 dpi; viral shedding was highest in younger animals during the early stage of viral infection, and of longest duration in the adult and old animals, particularly in the males of these groups. Organs appeared grossly normal and histopathological changes were generally mild on necropsy, except for one adult animal that had severe lung pathology.

#### 3.1.8 Poultry

Chickens, turkeys, ducks, quail, geese and pigeons were not able to be experimentally infected (Chen et al., 2020; Shi et al., 2020; Suarez et al., 2020) and did not seroconvert (Suarez et al., 2020), and are presumed to be not susceptible to SARS‐CoV‐2. The virus did not replicate in embryonated chicken eggs (Suarez et al., 2020).

A study by Chen et al. (2020) suggests a lack of co‐expression of ACE2 and the entry activator TMPRSS2 target receptors in lung cells of poultry, as well as mutations in critical amino acid sequences in ACE2 receptors, are responsible for the low susceptibility of these species to SARS‐CoV‐2.

#### 3.1.9 Pigs

Pigs were not able to be experimentally infected and are presumed to be not susceptible to SARS‐CoV‐2 (Shi et al., 2020). A study by Chen et al. (2020) reported that cells that co‐express ACE2 and TMPRSS2 receptors are targeted by SARS‐CoV‐2 for viral entry, and are widely distributed in a variety of porcine kidney and lung cells. The authors suggest that pigs may be able to function as intermediate hosts for SARS‐CoV‐2, despite contrary evidence of previous experimental studies, and invite further research on the subject.

### 3.2 Natural SARS‐CoV‐2 infection events in animals

The World Organisation for Animal Health (OIE) defined the following criteria for confirmed cases of SARS‐CoV‐2 in animals: isolation of SARS‐CoV‐2 from a sample taken directly from an animal, or identification of SARS‐CoV‐2 viral RNA in a sample taken directly from an animal that either targets at least two specific genomic regions at a level indicating the presence of infectious virus, or targets a single genomic region followed by sequencing of a secondary target (OIE, 2020e).

To date (July 2020), confirmed cases of SARS‐CoV‐2 in animals worldwide remain limited and sporadic, with global totals of fourteen domestic pets, eight captive big cats and an unreported number of mink (*Neovison vison*) on nineteen fur farms having definitively tested positive to SARS‐CoV‐2 (see Table 2). Most cases are strongly linked to close contact with confirmed or suspected human COVID‐19 patients, indicating that human‐to‐animal (anthroponotic) transmission is the primary mode of spread in domestic settings (Denis et al., 2020). In high‐density animal environments, including zoos and intensive breeding farms, subsequent between‐animal transmission has been presumed to occur, and at present there are two cases of probable animal‐to‐human transmission being investigated on Dutch mink farms. If confirmed, these cases would represent the first known cases of zoonotic transmission of the virus since the initial spillover event/s from bats to humans in China.

**TABLE 2:** Summary data for all animal cases (as defined by positive PCR test) of SARS‐CoV‐2 infection to date

Animal species	PCR positive	Virus isolation positive		Antibody positive		Clinical signs observed		Confirmed link to human case		Total confirmed infections
	*n*	*n*	%	*n*	%	*n*	%	*n*	%	*n*
Domestic										14
Dogs	3	1	33%	3	100%	1	33%	3	100%	3
Cats	11	0	0%	2	18%	8	73%	8	73%	11
Zoo										8
Tigers	5	DU	DU	DU	DU	4	80%	DU	DU	5
Lions	3	DU	DU	DU	DU	3	100%	DU	DU	3
Farmed										
Mink	19^†^	DU	DU	DU	DU	6^†^	32%	4	21%	19^†^

Abbreviations: DU, data unavailable.

^†^ Number of infected farms. Number of animals not reported.

Summary case data are presented below.

#### 3.2.1 Companion animals

##### Cats

Domestic cats are currently the most commonly reported companion animal species to be infected with SARS‐CoV‐2, with eleven confirmed cases reported from USA (*n* = 4), France (*n* = 2), Hong Kong (*n* = 1), Belgium (*n* = 1), Spain (*n* = 1), Germany (*n* = 1) and Russia (*n* = 1) to date (CDC, 2020; ENVT, 2020; FASFC, 2020; MAPA, 2020b; ProMED‐mail, 2020h; ProMED‐mail, 2020, 2020, 2020; WAHIS, 2020d). Of the pet cats to have tested positive for SARS‐CoV‐2, nearly all have had known (*n* = 8) or suspected (*n* = 2) close contact with human COVID‐19 cases. Infected cats have shown clinical signs in 8/11 cases, from mild upper respiratory disease including oral lesions and tongue ulceration (*n* = 1), fever (*n* = 2), sneezing and ocular discharge (*n* = 4), to moderate respiratory and/or gastrointestinal disease (*n* = 3). The Spanish cat was euthanized due to severe and deteriorating respiratory distress; however, its condition was attributed to underlying hypertrophic cardiomyopathy and the positive SARS‐CoV‐2 test finding was considered incidental (MAPA, 2020a). Of the remaining infected cats, nine have fully recovered either naturally or following supportive treatment; follow‐up data on the Russian cat have not been provided to date.

While most of the infected cats were from single‐cat households, the German case provided evidence of variable susceptibility to infection with and low transmission of SARS‐CoV‐2 between cats: three cats were resident in a retirement home that was experiencing a COVID‐19 outbreak, and only one tested positive, even after all three cats were subsequently quarantined together (ProMED‐mail, 2020j).

##### Dogs

Three dogs have tested positive for SARS‐CoV‐2 by PCR to date, all of which had had family exposure to COVID‐19 (WAHIS, 2020, 2020 WAHIS^2020^, ^2020^ ^2020^, ^2020^; WAHIS, 2020g). A 17‐year‐old Pomeranian in Hong Kong was the world’s first domestic pet to be confirmed with SARS‐CoV‐2 after testing positive in a quarantine facility on 26th February 2020. In total, the Pomeranian tested positive to SARS‐CoV‐2 by PCR over a period of 12 days, with higher viral loads and a longer duration of shedding in the dog’s nasal swabs than oral swabs. The dog had also developed antibodies to SARS‐CoV‐2 as indicated by a positive plaque reduction neutralization assay result. The dog died two days after its release from quarantine, reportedly due to an underlying geriatric disease (WAHIS, 2020a).

The other two canine cases were both German Shepherds, from Hong Kong and the USA. Both dogs had tested positive for SARS‐CoV‐2 antibodies, and virus was also isolated from the Hong Kong case (WAHIS, 2020h). The American case is the only dog to have reportedly showed signs of clinical respiratory disease; however, further details of clinical signs or disease severity were not provided (APHIS, 2020). Two additional dogs (the Netherlands, USA) tested antibody positive after their owners were confirmed to have COVID‐19 (Schouten, 2020a; WAHIS, 2020g). A pug in the USA was initially reported to have tested positive for SARS‐CoV‐2 during a university study, but negative results were obtained in subsequent confirmatory testing and the animal was eventually classified as negative (Fisher, 2020; Hauser & Gross, 2020).

#### 3.2.2 Zoo animals

A 4‐year old Malayan tiger *(Panthera tigris jacksoni)* at the Wildlife Conservation Society’s Bronx Zoo in New York developed mild clinical signs of respiratory disease (dry cough and some wheezing) from 27th March. Duplicate nasal and oropharyngeal swabs and tracheal wash samples were collected and confirmed to be positive for SARS‐CoV‐2 by RT‐PCR and sequencing on 4th April (WAHIS, 2020e). By 3rd April, three additional tigers (another Malayan and two Siberian tigers) from the zoo’s Tiger Mountain exhibit and three African lions from the African Plains exhibit were also showing similar respiratory signs, with anorexia also reported in one or more animals (no further details provided) (WCS, 2020a). Infection in these additional six symptomatic big cats and in another asymptomatic Siberian tiger also housed in Tiger Mountain was later confirmed using an rRT‐PCR test and genetic sequencing on opportunistically collected voided faecal samples, bringing the zoo’s total to eight cases (five tigers and three lions) (WAHIS, 2020, 2020; WAHIS^2020^, ^2020^ ^2020^, ^2020^). All eight animals in the two affected enclosures were isolated, and symptomatic animals were administered antibiotics and/or supportive care as needed. All were reported to be recovering well (WCS, 2020b).

As the zoo had been closed to the public since 16th March, transmission is presumed to have occurred from pre‐ or asymptomatic animal keeper/s infected with SARS‐CoV‐2 (WCS, 2020b), but no confirmed staff infections have been reported to date (ProMED‐mail, 2020g). It is also not confirmed whether all the cats were infected by the keeper/s, or whether there was subsequent between‐animal transmission. None of the other big cat species, including snow leopard, cheetah, clouded leopard, Amur leopard, puma or serval, nor any animals at the zoo have shown any signs of respiratory disease (Cordova, 2020; WCS, 2020b). Enhanced personal protective equipment (PPE) including surgical masks, face shields, gloves and coveralls, has been since been implemented for all staff caring for wild felid species in all zoos owned by the Society (WCS, 2020b).

#### 3.2.3 Farmed mink

##### The Netherlands

SARS‐CoV‐2 infections in farmed American mink (*Neovison vison*) were first reported on 26th April 2020, after “a few” mink on two Dutch fur farms showed respiratory and/or gastrointestinal disease, and increased mortality (Schouten, 2020b). At least one employee at each farm had reportedly shown signs consistent with COVID‐19 infection and subsequently tested positive for SARS‐CoV‐2 (Oreshkova et al., 2020). The farms housed 13,000 and 7,500 mink, respectively, most of which were pregnant females given the time of year. By late June, the total number of infected mink farms had risen to seventeen (Jonge & Schouten, 2020e; Jonge & Schouten, 2020f), all of which were located in North Brabant province, an area with high numbers of livestock farms (particularly poultry, pigs and mink) (ProMED‐mail, 2020e) and which at the time of the initial outbreaks was the country’s hardest hit region for COVID‐19 (Newmark, 2020).

Clinical signs of SARS‐CoV‐2 infections in mink, reported from four farms, ranged from mild watery nasal discharge to severe diffuse interstitial pneumonia and death. Overall adult mortality rates on the first and second farms were reported as 2.4% and 1.2% respectively, compared to a normal baseline mortality rate of 0.6%. No increase in kit mortality was observed. SARS‐CoV‐2 was detected by PCR in high concentrations in throat and nasal swabs from infected animals, and in conchae, liver and intestines (Oreshkova et al., 2020).

Genetic sequencing and phylogenetic analysis of viral samples from the first sampled mink suggest virus was introduced separately into the farms via an infected human, with subsequent between‐mink transmission at each location. Some companies own multiple farms, and in these cases, the infections are presumed to have been epidemiologically linked (Janssen, 2020; Schouten, 2020a). Non‐permeable cage separators prevent direct contact between mink; however, indirect transmission is presumed to have occurred via fomites, infectious aerosols and/or faecally contaminated dust particles (Oreshkova et al., 2020).

A national reporting requirement for any sign of respiratory disease and/or increased mortalities in farmed mink was introduced from 26th April (Schouten, 2020b). Protective measures were implemented on and around all infected farms, including visitor bans, transport bans for mink and mink manure, and PPE for farm employees. Public roads within a 400m radius of each premises were initially closed, but later reopened after testing of dust and air samples revealed that no virus was present outside the animal facilities (Schouten, 2020a). Additional surveillance and testing activities are described in the Surveillance section.

From 19th May, SARS‐CoV‐2 was designated an infectious animal disease in the Netherlands, with all mink companies required to submit the carcasses of mink that have died on their premises each week for “early warning” PCR testing (Jonge & Schouten, 2020b). Serological screening also required one‐off blood samples to be collected from all mink farms in the country, for antibody detection by ELISA. By late June 2020, an additional twelve infected premises had been detected by the “early warning” research (Jonge & Schouten, 2020d), bringing the country’s total number of infected farms, all of which will have to be depopulated, to seventeen.

Further research also indicated at least two “plausible” cases of mink‐to‐human infection (Schouten, 2020c), after genetic analysis of viral strains from infected employees revealed high homology with those in the farms’ infected mink, but not with environmentally derived samples or with virus from COVID‐19 patients in the region or even in the country (Denis et al., 2020; Jonge & Schouten, 2020a). The employees are presumed to have been infected by the mink before the outbreaks were evident, and prior to the implementation of PPE on the premises (Jonge & Schouten, 2020a).

From the 5th June, all infected mink farms were ordered to be depopulated in the interest of public health (Jonge & Schouten, 2020c), resulting in the euthanasia and disposal of over 500,000 mink by late June (HvN, 2020). The presence of peak numbers of mink kits on the farms at this time of year and their decreasing maternal antibody protection through the late summer months would increase the number of susceptible animals on‐farm by five to six times. The risk of uncontrolled viral transmission between mink and the potential for viral mutation and reassortment, as well as the establishment of a viral reservoir for ongoing human infections, was considered unacceptable (Jonge & Schouten, 2020c). Affected farms are to be cleaned, disinfected and quarantined following the cull. Owners will be compensated for the loss of their animals, and given the opportunity to permanently close their businesses ahead of the Dutch government’s mandatory ban on mink farming as of 1st January 2024 (Denis et al., 2020). The uninfected mink farms are still allowed to operate but are required to conduct ongoing surveillance activities (Jonge & Schouten, 2020c).

##### Denmark

The Danish Veterinary and Food Authority issued a press release on 17th June to advise that SARS‐CoV‐2 had been confirmed on a mink farm in the North Jutland region (MoF, 2020b). A second farm in the region was subsequently confirmed to be infected on 20th June (MoF, 2020a). Samples from mink on both farms were tested after one person associated with each farm was diagnosed with COVID‐19. On the second farm, the family dog was also reported to be positive for SARS‐CoV‐2 (MoF, 2020a). Entry and egress restrictions were placed on the mink farms while testing was being completed, and normal hygiene protocols for visitors to mink farms (including washing hands and changing clothes before and after animal handling) are still in force. The Danish government decreed that the infected farms will be depopulated in the interest of public health, and from 24th June implemented mandatory reporting of suspected or confirmed SARS‐CoV‐2 infections in Danish fur farms, and issued regulations for sampling and testing of fur animals (mink and ferrets), safe handling of feed and manure, and the quarantining, depopulation and disinfection of the infected premises (Denis et al., 2020; Larsen & Zuferov, 2020).

### 3.3 Surveillance of animal populations

#### 3.3.1 Testing of pets of human COVID‐19 patients

The 5th meeting of the OIE Advisory Group on COVID‐19 (OIE, 2020a) reported that routine testing of companion animals is being discouraged to “avoid concerned owners making unnecessary visits to their private veterinarian, which may increase public health risks through person to person contact”. It was also noted that “there have also been instances of humans submitting their own samples disguised as their pet’s sample to veterinarians in a desperate attempt to be tested”. The possibility of including a species probe in the diagnostic testing was discussed as a way of detecting this activity.

##### Hong Kong

Early in the pandemic, Hong Kong authorities took the unique step of routinely quarantining and testing companion animals owned by COVID‐19 patients (Denis et al., 2020). News reports suggested that as of 26th April, only the three known cases (two dogs and one cat as described in the case summaries) from 52 sampled pets were positive for SARS‐CoV‐2 (2/32 dogs, 1/18 cats and 0/2 hamsters) following PCR testing of nasal, oral and faecal samples (Cheng, 2020; Deacon, 2020). While the sample size is small, the infection prevalence of 5.8% among the tested animals is nevertheless of interest.

##### USA

The Center for Disease Control and Preparedness (CDC) has reportedly applied an ad hoc approach to companion animal testing in the USA, assessing pets of COVID‐19 patients on a case by case basis based on risk (OIE, 2020c). National Animal Health Laboratory Network (NAHLN) laboratories and a number of private laboratories are testing animal samples for SARS‐CoV‐2; however, there are reportedly only a small number of samples that have been tested so far (OIE, 2020c). All positive SARS‐CoV‐2 results obtained at NAHLN or private laboratories are confirmed at the United States Department of Agriculture Animal Plant Health Inspection Service’s National Veterinary Services Laboratory, with subsequent reporting to the OIE (OIE, 2020c). Media reports on 12th April suggested that the University of Illinois has tested gorilla, chimpanzee, cat, dog and armadillo samples, which all returned negative results (Knibbs, 2020).

The Washington State University’s Washington Animal Disease Diagnostic Laboratory (WADDL) commenced RT‐PCR testing of animal samples for SARS‐CoV‐2 in late March, with testing limited to animals linked to confirmed COVID‐19 patients, and experimental animals (WADDL, 2020a). Testing is conducted on nasal and oropharyngeal swabs collected by state‐approved veterinarians. Any PCR positives are re‐tested and partially genetically sequenced at WADDL, and submitted to the National Veterinary Services Laboratory for additional confirmatory testing. At the start of July 2020, 57 tests had been conducted on 25 cats, 2 tamanduas (a genus of anteater), 26 dogs, 2 ferrets, 1 camel and 1 mink, all of which were negative. Clinical signs were reported in some of the animals, including respiratory disease in the two tamanduas and two ferrets, all of which recovered; gastrointestinal disease in a dog that subsequently recovered; and sudden death in one cat that was due to hypertrophic cardiomyopathy as determined by autopsy (WADDL, 2020b).

Other university‐based research studies of pets and/or livestock belonging to COVID‐19 patients are reportedly being conducted in the New England region of the USA by Tufts University’s Cummings School of Veterinary Medicine—which is also collecting samples from animal owners (CUVM, 2020)—and in Canada by the University of Guelph’s Ontario Veterinary College (UoG, 2020).

##### Europe

Some laboratories in Italy are reportedly preparing to test pets (OIE, 2020b). The universities of Padua and Venice are reportedly planning serosurveys for SARS‐CoV‐2 in the small town of Vo, which was the epicentre of the initial outbreak in Italy, in order to assess the potential role of cats in the epidemiology of the disease (ProMED‐mail, 2020b; ProMED‐mail, 2020c). No routine testing of pets appears to be occurring in other European countries, although in Germany any animal that shows respiratory disease symptoms and has had contact with a COVID‐19 positive human is eligible for testing (Denis et al., 2020; OIE, 2020b). In the Netherlands, some companion animals of COVID‐19 patients with clinical disease appear to be being tested for SARS‐CoV‐2, as evidenced by a report of a positive test result in eight‐year‐old American bulldog that was euthanized due to severe deteriorating respiratory disease (Schouten, 2020a). Molecular diagnostic testing did not detect SARS‐CoV‐2; however, serological testing identified antibodies to SARS‐CoV‐2 in the dog’s blood. It was not reported whether the dog’s condition was attributed to SARS‐CoV‐2 or to another cause. The Dutch Agriculture Minister subsequently implemented requirements for veterinarians to report suspected SARS‐CoV‐2 infections in animals, and for laboratories to report positive test results, to the Netherlands Food and Consumer Product Safety Authority (Schouten, 2020a).

A French study by Temmam et al. (2020) tested 21 domestic animals (9 cats and 11 dogs) belonging to members of a veterinary student community. Eleven of eighteen students had clinical signs consistent with COVID‐19 (including fever, cough, anosmia etc), and two had tested positive to SARS‐CoV‐2 by RT‐PCR. While three cats displayed clinical signs consistent with coronavirus disease (respiratory or digestive signs), no pet tested positive for SARS‐CoV‐2 by RT‐PCR on nasal or rectal swabs and no animals demonstrated an antibody response when screened with an immunoprecipitation assay (Denis et al., 2020).

A preprint by Ruiz‐Arrondo et al. (2020) described an investigation of SARS‐CoV‐2 in companion animals taken from 17 households with confirmed human COVID‐19 patients in La Rioja, northern Spain. The 22 asymptomatic companion animals (12 dogs, 7 cats, 2 rabbits and 1 guinea pig) were quarantined, and oropharyngeal and rectal swabs were taken from each animal. An 8‐year‐old female domestic cat’s oropharyngeal swab was the only sample to test positive. A 7‐year‐old male domestic cat from the same household—whose owner was the only person in the study with severe COVID‐19 symptoms, requiring hospitalization—tested negative. Follow‐up nasopharyngeal and rectal swabs from both cats collected 26 days after the first samples all tested negative.

#### 3.3.2 Other surveillance activities

##### IDEXX surveillance and validation of novel SARS‐CoV‐2 RealPCR assay

IDEXX Laboratories have reportedly used their new “SARS‐CoV‐2 (COVID‐19) RealPCR Test” to perform an international surveillance study of SARS‐CoV‐2 in animals (IDEXX, 2020a; IDEXX, 2020b; IDEXX, 2020c). They have reported testing more than 5,000 specimens from dogs, cats and horses with respiratory disease in more than 17 countries (in North America, Europe and Asia). Over 3,500 specimens were reportedly sourced from all 50 states in the USA, and South Korea, from randomly selected diagnostic respiratory (77%, mostly deep pharyngeal and conjunctival) and diarrhoeal (23%, faeces) samples collected from canine (55%), feline (41%) and equine (4%) patients (IDEXX, 2020c). Locations with high community transmission at the time of sample collection (such as Seattle) were represented (Devlin, 2020; IDEXX, 2020c; ProMED‐mail, 2020a). Specimens were also tested in parallel with three assays from the CDC and all samples tested negative (Brulliard, 2020; IDEXX, 2020d; ProMED‐mail, 2020a). These results have been reported on IDEXX’s website but have not been peer‐reviewed, nor has a manuscript been released. Detailed metadata regarding sample type and location are not available. In the report, IDEXX state “Our monitoring of canine and feline specimens submitted for diagnostic respiratory RealPCR panels is ongoing and has now expanded to Canada, all US states, and countries within the EU, including areas with high rates of COVID‐19 in the human population” (IDEXX, 2020c).

##### Chinese testing of outbreak and archival animal samples

China reported to the OIE on 5th February 2020 that veterinary departments “had carried out 2019‐nCoV testing towards samples of pigs, poultry and dogs and other domestic animals collected since 2019 (mainly in late 2019)” (OIE, 2020d). So far, results of such testing are all negative. Prior to 9th February, the China Animal Health Epidemiology Center reportedly tested over 4,800 archived animal samples (including from poultry, cat, dogs and pigs) that had been collected in 2019 from numerous locations around China, all with negative results (OIE, 2020d; ProMED‐mail, 2020k). There is no indication that these samples included animals known or suspected to have had contact with COVID‐19 patients (ProMED‐mail, 2020k). Further reports exist that “animals from fur farms (including mink, foxes, raccoon dogs) have been tested for SARS‐CoV‐2 by RT‐PCR. So far all have been negative” (OIE, 2020b).

A preprint by Zhang et al. (2020a) reported results of serological screening performed on feline serum samples collected in Wuhan prior to and during the early stages of the outbreak. All samples taken before the outbreak (*n* = 39) were antibody negative, while 14.7% (15/102) of sera collected during the outbreak were antibody positive by ELISA, and confirmed by VNT and Western blot. Of the positive cats, three were owned by COVID‐19 patients, six were owned but had no known contact with COVID‐19 patients, and six were strays. Paired nasopharyngeal and rectal swabs collected from all cats during the study reportedly tested negative by RT‐PCR, indicating that no active infections were detected. The results suggest that the cats were infected by humans during the outbreak. A preliminary analysis of the transmission dynamics among these cats has estimated a R0 of 1.09 (95% confidence interval 1.05–1.13), indicating that sustained transmission between cats is unlikely to have occurred (Akhmetzhanov et al., 2020).

Another study from China reported testing for SARS‐CoV‐2 on 1,914 serum samples, collected at varying times and obtained from varying sources, from 35 animal species (including pigs, cows, sheep, horses, chickens, ducks, geese, experimental mice, rats, guinea pigs, rabbits, monkeys, dogs, cats, wild camels, foxes, minx, alpacas, ferrets, bamboo rats, peacocks, eagles, tigers, rhinoceroses, pangolins, leopard cats, jackals, giant pandas, masked civets, porcupines, bears, yellow‐throated martens, weasels, red pandas and wild boar) using a double‐antigen sandwich ELISA, with no positive results (Deng et al., 2020). Of most note is the lack of positive ELISA results among 15 pet and 99 street dogs from Wuhan (Deng et al., 2020). Otherwise, there is very limited information presented regarding the animal populations from which the samples were derived, and so very little inference can be made from this paper (Denis et al., 2020; Weese, 2020).

##### Mink farms in the Netherlands—including feral cats

Following the confirmed infections in several mink farms in the North Brabant region of the Netherlands in late April 2020, the Dutch Agriculture Minister initiated a series of surveillance activities in the country’s mink farms. Investigations on the infected farms included the testing of samples from sick and dead mink, manure and the environment; and genetic sequencing of viral isolates (ProMED‐mail, 2020, 2020; Schouten^2020^, ^2020^ ^2020^, ^2020^; ProMED‐mail, 2020f). Investigations into SARS‐CoV‐2 infections in domestic pets in the vicinity of infected farms, as well as in pigs, undomesticated farm cats and rabbits in the region, are also reportedly underway (Schouten, 2020d).

On the first two infected mink farms, serological surveillance of undomesticated farm cats revealed the presence of antibodies in 7/24 cats that were tested (Jonge & Schouten, 2020b; Schouten, 2020c). Of the positive cats, virus was detected in 1/7, however in too small a quantity to permit genetic sequencing (Jonge & Schouten, 2020a). At present, it is not known how or when the cats became infected, or what their role may have been in transmission of the infection between humans and mink on the farms, but the possibility of infection from mink is being investigated (Denis et al., 2020; ProMED‐mail, 2020l).

##### Great apes and other wild animal populations

While no natural or experimental cases of SARS‐CoV‐2 in great apes have been reported to date, their known susceptibility to common human respiratory viruses including rhinovirus C (the common cold) (Negrey et al., 2019) and the similarity of the ape form of the ACE2 receptor used by SARS‐CoV‐2 to infect human cells (Melin et al., 2020) has caused concern among primatologists and conservationists worldwide. Wildlife preserves across Africa and Asia have reportedly closed to the public to minimize human contact. Research teams in Tanzania, Cote d’Ivoire and Uganda, among others, are reportedly collecting faecal samples from wild gorillas and chimpanzees for virus testing, and training wildlife rangers to identify signs of respiratory distress in apes while also introducing PPE, quarantine and social distancing measures for in‐contact researchers (Gibbons, 2020). Use of PPE including face masks, gloves and coveralls was also recommended by the US National Wildlife Health Center for researchers conducting any wildlife handling or investigations into wildlife mortality events (Sleeman, 2020).

One predictive modelling study identified 291 bat species that are likely to be undetected hosts of betacoronaviruses, including 30 *Rhinolophus* species in addition to the 16 known hosts in this genus, suggesting that the potential bat reservoir for SARS‐CoV‐2 may be two‐thirds higher than currently described (Becker et al., 2020). A rapid risk assessment conducted by the US Geological Survey states a non‐negligible risk of human‐to‐bat transmission of SARS‐CoV‐2 in north America at present, but estimates that implementation of appropriate PPE including N95 respirator masks, dedicated outer clothing, and gloves, would reduce the exposure risk by 94%–96% (Runge et al., 2020). Should SARS‐CoV‐2 be introduced into the north American bat population, there would be an estimated 33.3% likelihood that the virus could spread and become established, thereby creating a potential reservoir of infection for humans and other animal species (Runge et al., 2020).

The OIE “EBO‐SURSY” project is reportedly proposing to test 3,000 samples previously collected from bats for haemorrhagic fever virus surveillance in West Africa for the presence of coronavirus, to investigate potential circulation of precursor viruses to SARS‐CoV‐2 (OIE, 2020b). American research institutes are also reportedly conducting infection studies in brown bats, to investigate the possibility of whether these wide‐ranging animals could become a reservoir for SARS‐CoV‐2 following human‐to‐bat transmission (OIE, 2020b), and surveillance of native north American wildlife generally to investigate their potential as reservoirs for SARS‐CoV‐2 (Sleeman, 2020).

## 4 DISCUSSION

While the initial SARS‐CoV‐2 spillover event is believed to have been a zoonotic transfer from bats to humans, possibly by way of an unidentified intermediate animal host, the driving force behind the pandemic has undeniably been human‐to‐human transmission (WHO, 2020). The vast number of human COVID‐19 patients worldwide and the incalculable numbers of human–animal interactions occurring each day make ongoing, unreported zoonotic and anthroponotic transmission of SARS‐CoV‐2 likely. The sheer number of human cases and the global shortages of adequately equipped and resourced diagnostic laboratories in recent months have prevented many countries from reaching testing targets in people; unsurprisingly, widespread testing or structured surveillance in animals has not been prioritized.

Nevertheless, there have been several cases of natural SARS‐CoV‐2 infections in animals confirmed globally. Cats are the most commonly reported domesticated animal to be infected, including both pet cats, and captive lions and tigers in a New York zoo. Pet dogs appear to be less susceptible to infection than cats, based on evidence from both the smaller number of reported cases and the results of experimental data. Most animal cases have had known or suspected exposure to human COVID‐19 patients, indicating that human‐to‐animal infection is the primary cause of spread in domestic settings. Nearly all infected animals have recovered naturally or following supportive treatments, except for those cases that died or were euthanized due to other underlying conditions. To prevent further anthroponotic transmission, animal owners have been advised to implement basic hygiene measures such as washing hands before and after contact with animals, their food or bedding, and to avoid cuddling, kissing or being licked by animals or sharing food with them. People who are suspected or confirmed to be infected with SARS‐CoV‐2 have been advised to limit contact with animals altogether (OIE, 2020f).

There is no evidence that people are at risk of contracting infection from their pets, and the OIE has advised that there is no justification in taking measures directed at companion animals that may compromise their welfare (OIE, 2020f). During the early stages of the pandemic, there were reports of stray dogs and cats rounded up in Russia (Balmforth, 2020), companion animals owned by confirmed COVID‐19 patients routinely euthanized by authorities in China, of large‐scale abandonment of pet dogs and cats and even of pets being thrown to their deaths from high‐rise buildings by fearful owners following early reports of pets testing positive for SARS‐CoV‐2 (Campbell, 2020). This occurred despite no evidence of animal‐to‐human transmission of the virus in domestic settings. In fact, the results of one modelling study predicted that abandoning cats could actually increase incidence of COVID‐19 in urban settings (Gao et al., 2020).

While SARS‐CoV‐2 infections in humans and animals within domestic settings are unlikely to contribute to community transmission, in high‐density animal environments, such as on farms with susceptible species, the risk of anthroponotic and zoonotic transmission must increase substantially. The lack of susceptibility of poultry and pigs to SARS‐CoV‐2 infection is reassuring, particularly given the heavy losses to these highly intensive commercial industries following recent and ongoing outbreaks of avian influenza and African swine fever, respectively (FAO, 2020b; Tian & Cramon‐Taubadel, 2020). Commercial farming of mink and ferrets, which are known to be susceptible to SARS‐CoV‐2 infection and replication (Kim et al., 2020; Richard et al., 2020; Schlottau et al., 2020; Shi et al., 2020), however, provides ideal conditions for viral transmission and spillover. The outbreaks of SARS‐CoV‐2 in Dutch and Danish mink farms are presumed to have been introduced by infected employees, followed by extensive mink‐to‐mink spread and potentially, mink‐to‐human transmission in at least two instances (Jonge & Schouten, 2020a; Larsen & Zuferov, 2020). Implementation of precautionary measures for all mink farms in these countries, including visitor bans and PPE for farm employees (MoF, 2020b; Schouten, 2020a), and the subsequent depopulation of all infected premises and the proper disposal of mink carcasses (Jonge & Schouten, 2020c; Larsen & Zuferov, 2020), will go some way to alleviating the occupational and public health risk posed by these farms.

However, despite the brief statement regarding negative testing results from Chinese fur farms (OIE, 2020b), there have been no reports of similar initiatives or surveillance activities being proactively implemented in other fur‐farming nations. The global fur trade was reported to be worth $40 billion in 2014, and while many countries have banned fur farming in recent years, the major producers in China, parts of Europe and North America reportedly continue to produce over 101 million animal pelts every year (Lung & Lin, 2019). China produced 26.2 million mink pelts or nearly 40% of the global mink pelt harvest in 2016 (Lung & Lin, 2019), while other major mink producers include Denmark (17.8 million), Poland (8.5 million), the Netherlands (5.5 million), the USA (3.5 million) and Canada (2.1 million) (Bale, 2016; Ingman, 2015; Lung & Lin, 2019). The high numbers of confirmed human COVID‐19 cases in these countries (Dong et al., 2020), coupled with particularly high densities of mink and other small mammals at this time of year due to seasonal breeding patterns (Amstislavsky et al., 2008), means it is possible that unreported SARS‐CoV‐2 infections are currently occurring in commercial fur farms. A recent report of asymptomatic experimental SARS‐CoV‐2 infections in rabbits (Mykytyn et al., 2020) may have implications for silent infections in commercial and smallholder rabbit farms across the globe—which in 2018 produced an estimated 920 million meat rabbits (FAO, 2020a) and 23 million kg of rabbit fur (Li et al., 2017)—and therefore warrants further investigation.

The potential for extensive virus transmission in these environments must be considered high. PPE use by farm workers can mitigate viral transmission, but only if it is consistently implemented. While the direct risk to the general public from these entities is low, increased community transmission can arise if on‐farm reservoirs of SARS‐CoV‐2 infections provide repeated spillover opportunities to naïve employees and their direct contacts.

Colonies of ferrets, non‐human primates and other potentially susceptible animals (including rabbits (Mykytyn et al., 2020)) kept for research purposes must similarly be at increased risk of introduction from infected humans, and could also provide reservoirs for ongoing viral replication, transmission and reverse spillover. Introduction of PPE and strict hygiene protocols for in‐contact researchers and animal support staff would be recommended to prevent occupational exposure to and transmission of the virus, and ongoing serological screening of both humans and animals could provide early warning of SARS‐CoV‐2 transmission within and between populations. Surveillance of commercial fur farms, colonies of research animals and other high‐intensity animal enterprises should be encouraged to further investigate their role in community transmission of SARS‐CoV‐2.

Of additional concern is the potential for anthroponotic transmission of SARS‐CoV‐2 to vulnerable wild animal populations. While no natural or experimental cases of SARS‐CoV‐2 in great ape species have been reported to date, their known susceptibility to common human respiratory viruses including rhinovirus C (the common cold) (Negrey et al., 2019), the similarity of their ACE2 receptors to the human form that SARS‐CoV‐2 uses to infect human cells (Melin et al., 2020), and the experimental studies that have demonstrated SARS‐CoV‐2 infections in other primate species (Lu et al., 2020; Rockx et al., 2020; Woolsey et al., 2020), suggest that SARS‐CoV‐2 is a tangible threat to great ape populations. Concern for highly vulnerable wild populations of great apes including chimpanzees and gorillas has already seen implementation of protective measures in wildlife preserves across Africa and Asia. Visitors have been banned from many such parks since early March 2020 to limit human contact with wild apes, and PPE, quarantine and social distancing measures for in‐contact researchers have also been implemented (Gibbons, 2020). Research teams in Tanzania, Cote d’Ivoire and Uganda, among others, are reportedly conducting observational and faecal surveillance of wild apes, and working with local communities to develop strategies for the further protection of great apes and wildlife, including the provision of goats, cash crops or other incentives to prevent villagers from hunting wild meat (Gibbons, 2020).

Other wild animal populations may also be at similar risk from SARS‐CoV‐2; however, it is too soon to know which populations are the most vulnerable. The virus has emerged so recently that few rigorous experimental studies about its infective potential in various animal species exist in the literature to date, and while numerous models have been developed to predict the susceptibility of animal species, large‐scale testing of these predictions would not be feasible for numerous reasons. Gryseels et al. (2020) argue that unless or until such evidence is obtained, sanitary precautions such as physical distancing and the wearing of PPE should be implemented during all human interactions with wild mammal species. This would serve to both protect individual animals from disease, and to prevent the establishment of SARS‐CoV‐2 reservoirs in wild animal populations, among which viral transmission could occur unchecked and potentially seed repeated spillover events into humans. This proposal is further strengthened by the US Geological Survey’s risk assessment regarding north American bats, which indicated that implementation of PPE for researchers could reduce the risk of human‐to‐bat transmission of SARS‐CoV‐2 by approximately 95% (Runge et al., 2020). The authors of this risk assessment identified several critical uncertainties that could affect their estimates of SARS‐CoV‐2 entering bat populations and associated sequelae, including likelihoods of human‐to‐bat, bat‐to‐bat, bat‐to‐animal and bat‐to‐human viral transmission dynamics; pathogenesis and replication of the virus in bat tissues; and seasonal impacts including bat breeding and hibernation patterns on virus replication and transmission (Runge et al., 2020). Until further research results are available to fill these knowledge gaps, a precautionary use of PPE and a conservative approach to wildlife handling and interaction would be encouraged.

Other adverse effects of SARS‐CoV‐2 on animals may include the “panic slaughter” of species that are mistakenly blamed for transmitting disease. The reputation of bats as the probable source of COVID‐19 has reportedly led to instances of fearful citizens setting fire to roosting bats (Fenton et al., 2020), and prompted calls for their mass slaughter to protect public health (Zhao, 2020). Even less extreme requests for the removal of hibernating bats from human residential areas could disturb their delicate physiological balances, lead to high bat mortality and potentially increase the spread of other viruses (Zhao, 2020). In addition, many bat species are endangered, and those that have become habituated to urban life may not survive in the wild, placing already fragile populations at further risk (Zhao, 2020). Masked palm civets (*Paguma larvata*) are widely accepted to have been the intermediate host species for SARS‐CoV‐1 (Gong & Bao, 2018), which led to the slaughter of thousands of wild civets in southern China using a variety of inhumane methods, including clubbing and drowning in disinfectant, following outbreaks in the region (Parry, 2004). Pangolins have been put forward as potential intermediate hosts for the current COVID‐19 pandemic (Andersen et al., 2020; Liu et al., 2020; Zhang, et al., 2020b), leading to concern from conservationists that pangolins, already endangered and reportedly the world’s most trafficked mammal, may be placed at further risk by “ecological killing” as part of disease control efforts (Standaert, 2020). On the other hand, the zoonotic origins of the COVID‐19 pandemic have increased global calls to end the trading of wildlife for all purposes, including “wet” animal markets and wild game hunting for human consumption, and the traditional medicine, tourism, wild pet and fur farming sectors still existing across the globe. In March 2020, China introduced a ban on hunting, trading, transporting and eating the meat of wild animals (NPC, 2020) and in early June upgraded the protection of all pangolin species to the highest level, banning all trade in pangolins and their products and removing pangolin scales from their approved list of traditional medicines (Yueming, 2020). While this is to be applauded, China’s reported proposal to reclassify mink, raccoon dogs, and silver and blue foxes from wild animals to domestic livestock (Dalton, 2020) may cause additional concerns, both for animal activists, and public health advisors concerned about extended SARS‐CoV‐2 transmission and the potential for future zoonotic pandemics.

Direct impacts of the COVID‐19 pandemic on animals have been mostly limited to date, with mild to moderate transient clinical disease reported in less than half of the 14 pets and 8 zoo animals confirmed to be infected. Nevertheless, the pandemic has negatively impacted animal welfare directly in several reported instances—including the fatalities and depopulation of hundreds of thousands of farmed mink, and the dogs and cats that have been abandoned and thrown out of high‐rise buildings—and most likely indirectly, in many more unreported instances, such as via decreased activities of veterinary services during global lockdown (Gortázar & Fuente, 2020). While animals are not implicated in community transmission of SARS‐CoV‐2 at present, until more data from natural cases, surveillance and experimental infection studies become available, the role of animals in SARS‐CoV‐2 transmission remains uncertain. Should SARS‐CoV‐2 become established in domestic or wild animal populations, the potential impacts of the resulting panzootic could be severe: morbidity and mortality in susceptible animals of high emotional, economic and/or agricultural value; threats to the health and survival of vulnerable wildlife species; and establishment of viral reservoirs that could seed repeated spillover events into humans and thwart global disease control or eradication efforts. Continuing research and surveillance activities are needed, to further determine the role of animals in community transmission of SARS‐CoV‐2; to advise the public to prevent fear, ignorance and misinformation that may cause adverse animal welfare events; and to identify the reservoir and intermediate (if applicable) hosts of SARS‐CoV‐2 so that future spillover events can be prevented.

## DISCLOSURE STATEMENT

Please note that similar sections of this paper have been previously published by the authors in the “Covipendium”, a non‐peer‐reviewed living paper curated by the Rega Instituut, KU Leuven, Belgium (Denis et al., 2020).

## CONFLICT OF INTEREST

The authors have no conflicts of interest to declare.

## ETHICAL APPROVAL

The authors confirm that the ethical policies of the journal, as noted on the journal’s author guidelines page, have been adhered to. No ethical approval was required as this is a review article with no original research data.

## Data Availability

Data sharing is not applicable to this article as no new data were created or analysed in this study.
